# High-Resolution Melting Analysis as a Powerful Tool to Discriminate and Genotype *Pseudomonas savastanoi* Pathovars and Strains

**DOI:** 10.1371/journal.pone.0030199

**Published:** 2012-01-25

**Authors:** Andrea Gori, Matteo Cerboneschi, Stefania Tegli

**Affiliations:** Laboratorio di Patologia Vegetale e Molecolare, Dipartimento di Biotecnologie Agrarie, Università degli Studi di Firenze, Sesto Fiorentino, Firenze, Italy; Nanjing Agricultural University, China

## Abstract

*Pseudomonas savastanoi* is a serious pathogen of Olive, Oleander, Ash, and several other Oleaceae. Its epiphytic or endophytic presence in asymptomatic plants is crucial for the spread of Olive and Oleander knot disease, as already ascertained for *P. savastanoi* pv. *savastanoi* (*Psv*) on Olive and for pv. *nerii* (*Psn*) on Oleander, while no information is available for pv. *fraxini* (*Psf*) on Ash. Nothing is known yet about the distribution on the different host plants and the real host range of these pathovars in nature, although cross-infections were observed following artificial inoculations. A multiplex Real-Time PCR assay was recently developed to simultaneously and quantitatively discriminate *in vitro* and *in planta* these *P. savastanoi* pathovars, for routine culture confirmation and for epidemiological and diagnostical studies. Here an innovative High-Resolution Melting Analysis (HRMA)-based assay was set up to unequivocally discriminate *Psv*, *Psn* and *Psf*, according to several single nucleotide polymorphisms found in their Type Three Secretion System clusters. The genetic distances among 56 *P. savastanoi* strains belonging to these pathovars were also evaluated, confirming and refining data previously obtained by fAFLP. To our knowledge, this is the first time that HRMA is applied to a bacterial plant pathogen, and one of the few multiplex HRMA-based assays developed so far. This protocol provides a rapid, sensitive, specific tool to differentiate and detect *Psv*, *Psn* and *Psf* strains, also *in vivo* and against other related bacteria, with lower costs than conventional multiplex Real-Time PCR. Its application is particularly suitable for sanitary certification programs for *P. savastanoi*, aimed at avoiding the spreading of this phytopathogen through asymptomatic plants.

## Introduction

*Pseudomonas savastanoi* is a phytopathogenic bacterium, causal agent of Olive (*Olea europaea* L.) and Oleander (*Nerium oleander* L.) knot disease. Isolates from Ash (*Fraxinus excelsior* L.) are also included in this species, whose taxonomy and classification have been controversial for a long time. According to several phenotypic, physiological, biochemical, and molecular parameters, isolates from Olive, Oleander and Ash are now accepted as belonging to distinct pathovars, named *P. savastanoi* pv. *savastanoi* (*Psv*), pv. *nerii* (*Psn*) and pv. *fraxini* (*Psf*), respectively [1]–[18]. Actually, under experimental conditions the host range of these pathovars was shown to be wider than what it could be assumed according to the concept of pathovar: for example *Psn* was demonstrated to be capable of infecting Olive as well as Ash [7], and *Psf* can multiply in Olive bark when artificially inoculated [5]. But in natural environments their distribution on the different hosts and the occurrence of cross-infections are still unknown. On the contrary, the spread of the disease was already assessed to be closely related to the diffusion and the amount of *P. savastanoi* populations, both surviving within the young knots on diseased and symptomatic plants, and naturally resident as epiphytes on healthy and/or asymptomatic plants [19]–[25]. Weather conditions conducive for both *Psv* epiphytic development and its entry into the host strictly determine the extent of damages caused by *Psv* [21]–[22], [24]–[29]. For this reason, all the traditional control strategies applied up to now to Olive knot disease are mainly targeted on reducing the endophytic and epiphytic *P. savastanoi* populations, with practices such as pruning of affected branches and the use of copper compounds. But today pruning is not such a common practice as it was in the past because of the high costs, and the effectiveness of conventional copper compounds had a sharp drop for the spread of copper-resistant isolates among *P. syringae* pathovars and related bacteria [30]–[31]. Thus to avoid the spread of *P. savastanoi* knot diseases, sanitary certification programs for Olive and Oleander propagation materials and mother plants were already launched worldwide [32]–[34]. Unfortunately, these measures are still based exclusively on the visual inspection of the typical hyperplastic knots, without even considering the possible asymptomatic presence of *P. savastanoi* [19]–[20], [23]–[25].

Conventional microbiological assays for *Psv* detection and identification have been available for two decades, but they are time consuming and they have very low sensitivity and specificity [35]–[36]. Therefore, several PCR-based protocols were developed for the rapid and sensitive detection of *Psv* and *Psn*, also working *in planta* and on asymptomatic tissues [37]–[40]. However, all these assays absolutely failed to differentiate *Psv*, *Psn* and *Psf* isolates. This important limit for their routine applicability in sanitary certification programs was recently overcome by the development of a pathovar-specific, highly sensitive and quantitative Real-Time PCR procedure [41]. However a similar approach requires a labeled probe for each target to be identified, which results in a substantial increase in cost. Currently less expensive identification methods are going to be preferred, such as those using common and widely available reagents, along with unlabeled oligonucleotides and a high saturating double strand DNA (dsDNA) binding dye. To this end, High-Resolution Melting Analysis (HRMA) provides a rapid, simple, high-throughput, cost-effective, and alternative single-tube approach to the direct DNA sequencing for the detection of single-nucleotide polymorphisms (SNPs). These mutations are highly informative of genotypic variations which have discriminative potential and are particularly useful in analyzing a large number of samples [42]–[44]. In the last few years, this novel technique has been investigated and successfully applied also for diagnostic purposes in many different research areas, as far as detection and analysis of cancer-related mutations in humans [45]–[46], identification and genotyping of parasites and pathogens of animals and humans [47]–[51], and plant genotyping [52]–[57].

Here we report the development of a unique strategy, based on Real-Time PCR followed by HRMA, for the rapid, highly specific, and sensitive detection and identification of *Psv*, *Psn*, and *Psf.* To our knowledge this is the first report of a HRMA-based assay developed so far for a plant pathogenic bacterium, as well as one of the few multiplex-HRMA protocols [58]–[59]. Moreover, this assay offers the opportunity to easily and simultaneously discriminate and genotype the pathovars *Psv*, *Psn* and *Psf*, *in vitro* as well as *in planta*. This novel application of HRMA would be extremely helpful in epidemiological studies aimed to unveil the distribution in nature of these *P. savastanoi* pathovars on the different host plants, and to deeper investigate the genetic structure of this species, as well as to design innovative and more efficient control measures for Olive and Oleander knot disease, starting from sanitary certification programs.

## Materials and Methods

### Bacterial strains and growth conditions

The *P. savastanoi* strains used in this study, isolated from different hosts and having various geographical origins, are reported in Table 1. Several bacteria closely related to *P. savastanoi* or ubiquitous on plants, water and soil were also tested (Table S1). They were routinely grown at 26°C, in King's B (KB) medium [60] broth or agar. Bacterial growth was monitored by determining the optical density at 600 nm (OD_600_) at different times during incubation, and bacterial concentrations were estimated by serial dilutions and plate counts. For long-term storage, bacteria were maintained at −20°C and −80°C on 40% (v/v) glycerol.

**Table 1:** *P. savastanoi* strains used in this study with the designation of their geographical origin, the variants for each SNP marker examined and their SNP-group.

Strain^a^	Geographical origin	SNP markers										SG^c^
		S	J	C1	C2	V	R	JL1	JL2	L1	L2	
***Psv***												
LPVM510	Southern Italy	C	G	T	A	G	G	C	T	G	A	I
LPVM602		C	G	T	A	G	G	C	T	G	A	I
LPVM702		C	G	T	A	G	G	C	T	G	A	I
PVBa223		C	G	T	A	G	G	C	T	G	A	I
LPVM422		C	G	T	A	G	G	C	T	G	A	I
ES47		C	G	T	A	G	G	C	T	G	A	I
ITM KL1	Central Italy	C	G	T	A	G	G	C	T	G	A	I
ITM KS1		C	G	T	A	G	G	C	T	G	A	I
MC41		C	G	T	A	G	G	C	T	G	A	I
MC72		C	G	T	A	G	G	C	T	G	A	I
MC1		C	G	T	A	G	G	C	T	G	A	I
LPVM20		C	G	T	A	G	G	C	T	G	A	I
MC33		C	G	T	A	G	G	C	T	G	A	I
LPVM15		C	G	T	A	G	G	C	T	G	A	I
MC66		C	G	T	A	G	G	C	T	G	A	I
MC80		C	G	T	A	G	G	C	T	G	A	I
LPVM8		C	G	T	A	G	G	C	T	G	A	I
ESB28	Southern Italy	T	G	G	A	T	G	C	T	G	A	II
ESB35		T	G	G	A	T	G	C	T	G	A	II
ITM317^b^		T	G	G	A	T	G	C	T	G	A	II
ESB49		T	G	G	A	T	G	C	T	G	A	II
ESB50		T	G	G	A	T	G	C	T	G	A	II
LPVM5-2		C	G	G	A	T	G	C	T	G	A	III
LPVM1		C	G	G	A	T	G	C	T	G	A	III
LPVM1-2		C	G	G	A	T	G	C	T	G	A	III
LPVM2		C	G	G	A	T	G	C	T	G	A	III
LPVM3		C	G	G	A	T	G	C	T	G	A	III
LPVM3-2		C	G	G	A	T	G	C	T	G	A	III
ES31	USA (California)	C	G	G	A	T	G	C	T	G	A	III
ES32		C	G	G	A	T	G	C	T	G	A	III
ES34		C	G	G	A	T	G	C	T	G	A	III
***Psn***												
ESC24	USA (California)	C	G	T	C	G	T	C	C	G	A	IV
ESC36		C	G	T	C	G	T	C	C	G	A	IV
ESC43^b^	Southern Italy	C	G	T	C	G	T	C	C	G	A	IV
ESC45		C	G	T	C	G	T	C	C	G	A	IV
ESC8		C	G	T	C	G	T	C	C	G	A	IV
ESC81		C	G	T	C	G	T	C	C	G	A	IV
ESB60		C	G	T	C	G	T	C	C	G	A	IV
ESB15		C	G	T	C	G	T	C	C	G	A	IV
LPVM71		C	G	T	C	G	T	C	C	G	A	IV
LPVM33		C	G	T	C	G	T	C	C	G	A	IV
LPVM39-1		C	G	T	C	G	T	C	C	G	A	IV
LPVM39-2		C	G	T	C	G	T	C	C	G	A	IV
NCPPB640	Ex Yugoslavia	C	G	T	C	G	T	C	C	G	A	IV
***Psf***												
AG35	Italy	C	A	G	A	T	G	T	T	A	G	V
PD120-1	The Netherlands	C	A	G	A	T	G	T	T	A	G	V
LPVM16		C	A	G	A	T	G	T	T	A	G	V
LPVM17		C	A	G	A	T	G	T	T	A	G	V
AG51	France	C	A	G	A	T	G	T	T	A	G	V
NCPPB1464	United Kingdom	C	A	G	A	T	G	C	T	A	G	V
MCA1	Italy	C	A	G	A	T	G	C	T	A	G	VI
MCA2		C	A	G	A	T	G	C	T	A	G	VI
MCA3		C	A	G	A	T	G	C	T	A	G	VI
MCA4		C	A	G	A	T	G	C	T	A	G	VI
CFBP1838	France	C	A	G	A	T	G	C	T	A	G	VI
NCPPB1006^b^	United Kingdom	C	A	G	A	T	G	C	T	A	G	VI

a CFBP, Collection Française de Bactéries Phytopathogènes, INRA, Angers, France; ITM, Culture collection of Istituto Tossine e Micotossine da Parassiti vegetali, C.N.R., Bari, Italy (from A. Sisto); LPVM, Culture Collection of Laboratorio di Patologia Vegetale Molecolare, Dipartimento di Biotecnologie Agrarie, Università degli Studi di Firenze; NCPPB, National Collection of Plant Pathogenic Bacteria, York, UK (http://www.nctc.org.uk); PD, Culture collection of Plant Protection Service, Wageningen, The Netherlands; PVBa, Culture Collection of Dipartimento di Patologia Vegetale, Università degli Studi di Bari, Italy (from A. Sisto); ES, MC and AG, from E. Santilli, M. Cerboneschi and A. Gori, respectively.

b Code used at LPVM for strains ITM317, NCPPB1006 and ES23 are *Psv5*, *Psf134* and *Psn23*, respectively.

c SG = SNP-group.

Contaminations were periodically monitored by 16S rDNA amplification followed by enzymatic restriction with *AluI* [15], [61], and by *P. savastanoi* PCR specific assays [41].

### Molecular techniques

Genomic DNA was extracted and purified from bacterial liquid cultures at OD_600_ = 0.8, using Gentra® Puregene kit (Qiagen, Valencia, CA, USA), according to manufacturers' instructions. DNA concentration was evaluated both spectrophotometrically, with NanoDrop™ ND-1000 (NanoDrop Technologies Inc., DE, USA), and visually by standard agarose gel electrophoresis [1% agarose (w/v) in TBE 1×] [62]. Genomic DNA to be immediately used in conventional PCR was also obtained by direct lysis of single bacterial colonies, picked up from overnight agar plate cultures, carefully resuspended in sterile distilled water (100 µl for each colony), incubated at 95°C for 10 min and immediately cooled on ice. After a spin in a microcentrifuge (10,000 *g* for 10 min) to pellet cell debris, 2.5 µl lysate was directly used in PCR assays as template.

DNA amplicons were extracted and purified from agarose gel with PureLink® Quick Gel Extraction Kit (Invitrogen Inc., Carlsbad, CA, USA), under the conditions recommended by the manufacturer, and double-strand sequenced at Eurofins MWG Operon Ltd (Ebersberg, Germany). Multiple sequence alignments and comparisons were performed using the computer package CLUSTALW (version 2, http://www.ebi.ac.uk/Tools/clustalw2) [63], and by means of Basic Local Alignment Search Tool (BLAST, http://www.ncbi.nlm.nih.gov/blast) [64].

### Real-Time PCR and HRM analysis

Primer pairs to be used in HRM analysis were designed according to the sequencing of the entire Type Three Secretion System (TTSS) clusters of three representative *P. savastanoi* strains *Psv*5, *Psn*23 and *Psf*134 (Accession Numbers FR717896, FR717897, FR717898, respectively) [65], using Beacon Designer 7.5 software (Premier Biosoft International, Palo Alto, CA, USA) (Table 2; Figure S1).

**Table 2:** Details and performances of the nine primer pairs used in this study for the identification and genotyping of *P. savastanoi* by HRMA.

Primer	Sequence (5′ to 3′)	Amplicon size (bp)	SNP Marker	SNP sequence (5′ to 3′)^a^	Amplicon Tm (°C)	Pre-melt^b^ (°C)	Post-melt^b^ (°C)	LLOD^c^ (fg)
hrpS_for	AGCGGCACAAGGCGGAAC	156	S	…ATCGA(**T/C**)GATCA…	87,2–87,6	85,7–86,0	88,6–89,0	10
hrpS_rev	TGGGCCGAAGCGATCACG							
hrpJ_for	CAACCAGCGACATGAACC	102	J	…GCCAC(**G/A**)CCAGT…	88,6–87,8	84,7–85,2	90,5–91,0	1
hrpJ_rev	TGACCCCTTCTTTCTTGAAG							
hrcC1_for	GCCTTGATCGGGTGTTGA	97	C1	…ATCCG(**T/G**)GAAAA…	85,0–85,4	82,2–82,7	87,3–87,8	100
hrcC1_rev	CGCACATCGGCTGTTACA							
hrcC2_for	CGCCAGCCACAGATATCG	103	C2	…GAGCG(**A/C**)AACCA…	82,4–83,0	79,5–80,0	84,7–85,2	10
hrcC2_rev	AACAGCACCAGCACAACT							
hrpV_for	CGTCCCGAGCAACTGAGAGAG	162	V	…ACTAC(**T/G**)TTCTG…	85,4–85,8	82,9–83,4	87,1–87,6	1
hrpV_rev	ATGTCGCCGTATGTCATCCAGG							
hrcR_for	CACCTGCTGAACGCCAATT	113	R	…CAACA(**G/T**)AAAGG…	83,6–84,2	81,0–81,5	85,7–86,2	100
hrcR_rev	TGTTTCTCGGCTCGCTGTC							
ncJL_for	GGTTCTGAGCCTGGTCAT	112	JL1 and LJ2	…TG(**C/T**)CAAAAGC(**T/C**)GT…	83,8–84,8	81,4–81,9	86,5–86,0	10
ncJL_rev	CGCAACGCCGTTTTTATC							
hrpL1_for	GTCAACTGACGGCTGATC	106	L1	…TTCAG(**G/A**)GCTGG…	83,2–83,6	80,7–81,2	85,0–85,5	10
hrpL1_rev	GCCTCCAGAAATACGCATT							
hrpL2_for	AGCCGCAGACCTGGTTGTG	159	L2	…CATTC(**A/G**)GAAGC…	81,2–81,6	79,0–79,6	82,9–83,5	1
hrpL2_rev	ATTGCCTGTGCCCGTCTACC							

a In bold the two variants for each SNP marker are shown.

b Pre- and post-melt temperatures used to normalize HRM data.

c LLOD = Lower Limit Of Detection.

Real-Time PCR was carried out on a CFX96 cycler – RealTime PCR Detection System (Bio-Rad Laboratories, Inc., Hercules, CA, USA), in white-walled PCR plates (96 wells), with 10 µl reaction mixture containing 0.5 µM of each primer, 1× SsoFast™ EvaGreen® Supermix (Bio-Rad Laboratories, Inc.) according to manufacter's instructions, and as template 10 ng of genomic DNA, unless otherwise specified. Cycling conditions were one cycle at 95°C for 5 min, followed by 40 cycles at 95°C for 5 s and 60° for 5 s. The amount of fluorescence for each sample, given by the incorporation of EvaGreen® into dsDNA, was measured at the end of each cycle and analyzed via CFX-Manager Software v1.6 (Bio-Rad Laboratories, Inc.). Melting curves of PCR amplicons were obtained with temperatures ranging from 65°C to 95°C. Data acquisition was performed for every 0.2°C increase in temperature, with a 10 s step. For each strain three batches of DNA were obtained from separate extractions performed on different bacterial cultures. Each DNA batch was then tested in three independent experiments, and analyzed by High-Resolution Melting analysis software (Bio-Rad Laboratories, Inc.), which automatically clusters the samples according to their melting profiles and assigns a confidence score to each sample. The confidence level threshold for a sample to be included in a cluster was 99.5%. HRM curves were normalized using the pre- and post-melting temperatures of each primer pair (Table 2), automatically given by the software or manually adjusted to achieve better results. This would minimize the variations in fluorescence magnitude among samples, in order to analyze and compare the curve profiles deriving from the strains tested here [66]. Derivative plots were also generated to assess the different melting peaks. The lower limit of detection (LLOD) for each primer pair was evaluated by using different amounts of DNA template (from 10 ng to 1 fg per reaction): LLOD was the lowest DNA amount giving reliable and reproducible results in all the three replicates of different PCR runs.

### HRMA-based strains clustering and phylogenetic analysis

The HRM data were confirmed by sequencing the amplicons obtained. The distribution of the variants for each SNP marker was also assessed for all the 56 *P. savastanoi* strains tested here. Each strain was then assigned to one of the six distinct SNP-groups (SGs), according to its specific “SNPs-code”, given by the concatenated bases for each of the ten SNPs analyzed, strictly ordered according to their real distribution on TTSS cluster (Table 1). Genetic distances among the 56 *P. savastanoi* strains were also calculated, using their SNPs-code as input file for MEGA software (version 5.01) [67]. An UPGMA (Unweighted Pair Group Method with Arithmetic Mean) [68] tree was produced by the p-distance method [69]. Bootstrap values were computed by MEGA with 1,000 replications, to evaluate the significance of the branching order [70].

### HRM analysis on *P. savastanoi* from infected Oleander plants

The possibility of cross-reaction events during HRM analysis on *P. savastanoi* with bacterial epiphytes residing on Oleander plants was *in vivo* evaluated. Leaves taken from adult Oleander plants (3 years old) were inoculated with a drop of a bacterial suspension of *P. savastanoi* in sterile saline water (SSW, NaCl 0.85% in distilled water) with an OD_600_ = 0.5 (corresponding to about 0.5×10^8^ CFU/ml), on the upper or on the lower page of the leaf. Different bacterial drop volumes (5, 10, 20, 50 or 100 µl) were tested. The strains used were *Psv*5, *Psn*23 and *Psf*134. For each treatment three replicates were performed in three independent experiments. Leaves spotted with SSW were used as negative controls. The inoculated leaves were then incubated in a moist sterile chamber, made in a Petri dish, for 30 min at room temperature. After that time each drop was separately recovered washing the leaf with 100 µl of SSW. The leaf washings were separately centrifuged at 10,000 *g* for 5 min, resuspending then the pellets in sterile water to be used for thermal lysis DNA extraction. As PCR template, 2.5 µl of thermal lysis solution were directly used in each reaction. Serial dilutions of pure genomic DNA (from 1 ng to 1 pg/reaction) from strains *Psv5, Psn23* and *Psf134* were used as positive controls to confirm the identity of each sample tested by comparing its profile to that of the closest DNA dilution (+/−2 Ct, assessed during amplification phase).

In order to ascertain the composition of the bacterial microflora epyphitically resident on the Oleander leaves sampled here, a global approach capable of identifying both *in vitro* culturable and unculturable bacteria was adopted. On leaf washings from negative controls, amplification of 16S rDNA was carried out [15], [61], then used for cloning procedure, Amplified rDNA Restriction Analysis (ARDRA) [61], and 16S rDNA sequencing.

The HRMA-based protocol developed here was also tested on *in vitro* micropropagated Oleander plants (Vitroplant Italia s.r.l., Cesena, Italy) artificially infected with strain *Psn23*. Oleander plants were grown for 3 weeks at 26°C on Murashige-Skoog medium [71] without addition of any phytohormone, with a photoperiod of 16 h/light-8 h/dark. After that time Oleander plants were wounded on the stem at the second internode, using a 1 ml syringe needle, and immediately inoculated with 1 µl of a bacterial suspension in SSW having an OD_600_ = 0.5. Negative control plants inoculated with SSW alone were also included. The infected plants were grown in the same conditions as those described above. Fourteen days after inoculation, stems (about 100 mg each) were separately removed, aseptically and finely chopped with a scalpel, and soaked for 30 min in 6 ml of SSW, with shaking (100 rpm). After that time, any plant material was removed with a sterile filter gauze, and the filtrate was centrifuged at 12,000 *g* for 5 min. To each bacterial pellet, 100 µl of sterile water was added. The suspension was directly thermal lysed for DNA extraction, and then used as template in HRM-PCR, as such or ten-fold diluted. The identity of each sample was confirmed as described above.

## Results

### Real-Time PCR and HRM analysis

Successful amplifications were achieved for all the 56 *P. savastanoi* strains tested (Table 1), using the nine primer pairs designed on the basis of the TTSS sequences of the three representative *P. savastanoi* strains *Psv5*, *Psn23* and *Psf134* (Figure S1). Most of the SNPs identified on the alignment of the TTSS sequences of these strains were transition mutations (T to C or C to T, A to G or G to A) (60%), but transversion mutations (the substitution of a purine for a pyrimidine or vice versa) were also included.

Since no information was available at that time about the TTSS sequences of the other 53 *P. savastanoi* strains, these bacteria could be considered blind-tested.

For each amplicon, normalized, difference, and derivative melt plots were produced.

**Figure 1:**
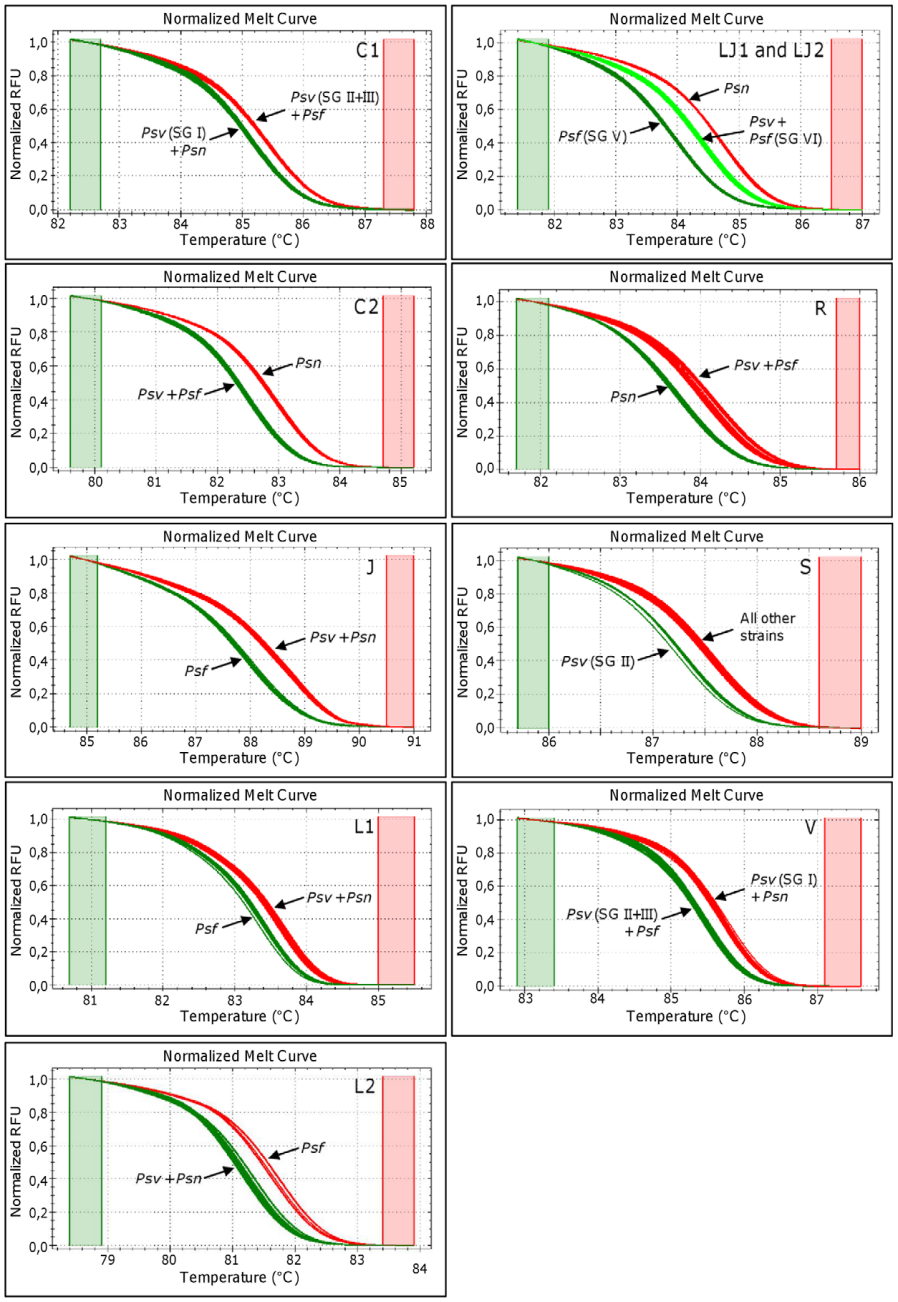
Normalized HRM plots related to the ten SNPs analyzed in this study. Normalized HRM plots of the amplicons obtained from the 56 *P. savastanoi* strains using the nine primer pairs. For each amplicon, the corresponding SNP marker is reported on the upper-right corner of the plot. Different colors are used to indicate distinct profiles, corresponding to the HRMA-based clustering of the 56 *P. savastanoi* strains into pathovars or SGs. RFU: Relative fluorescence units. Green and red columns represent pre- and post-melting normalization regions.

The HRM analysis on normalized melting and difference curves always distributed the 56 *P. savastanoi* strains into two or three independent clusters, readily and consistently resolved (Figure 1; Figure S2). Surprisingly, some SNP markers did not allocate together all the strains belonging to *Psv* or to *Psf*, as it should be occurred assuming the SNPs found in the TTSS of *Psv5* and of *Psf134* characterizing the pathovar they belong to. On the contrary *Psn* strains were never splitted in more than one cluster.

Therefore all the amplicons were sequenced in both strands. The sequencing confirmed and perfectly explained the HRM data obtained. According to the alignment among the TTSS sequences of *Psv5*, *Psn23* and *Psf134*, a single SNP appeared to be present in each amplicon (Figure S1). Actually, the sequencing of the HRM-amplicons corresponding to the non-coding region between *hrpL* and *hrcJ* revealed two separate SNPs (then named JL1 and JL2), differently distributed among the *Psf* strains tested here, instead of the single one that was expected (JL2) (Table 1; Figure S1). Similarly the HRM clustering obtained for *Psv* strains was confirmed by the sequencing, showing intra-pathovar variants for the SNP markers C1, S and V (Table 1). On the contrary, the SNP variants found on TTSS sequence of *Psn23* were perfectly matching the amplicon sequencing results obtained from the other *Psn* strains.

According to these findings, no SNP marker among those tested was able to produce a cluster exclusively including all the *Psv* strains here assayed. Only five out of these ten SNP markers were able to exclusively group together *Psn* and *Psf* strains, but could not simultaneously distinguish the three pathovars from each other (Figure 1; Figure S2). In particular, the SNP markers C2 and R discriminated all the *Psn* strains from those belonging to the other *P. savastanoi* pathovars, as well as markers L1, L2 and J characterized all the *Psf* strains. On the contrary, the remaining SNP markers always grouped together strains belonging to different pathovars. As far as SNP marker S is concerned, just the five *Psv* strains belonging to the SG II were grouped together, unlike a composite group given by the remaining *Psv* strains (SGs I and III) and all the strains belonging to *Psn* and *Psf.* The markers C1 and V grouped the *Psv* strains belonging to SG I together with all the *Psn* strains, and the others (SGs II and III) with *Psf* strains. The amplicon including the two SNP markers JL1 and JL2 produced three different melting curves. One cluster was exclusive for *Psn* strains, one included the *Psf* strains belonging to SG IV, and another one clustered the remaining *Psf* strains (SG V) with those belonging to *Psv* (Figure 1; Figure S2).

### HRMA-based *P. savastanoi* pathovar genetic diversity

The 56 *P. savastanoi* strains tested were each assigned to one of the six different SGs, identified according to the possible combinations that were obtained among the SNPs variants, revealed by HRM runs and then assessed by HRM amplicons sequencing. Each SG always included only strains belonging to the same pathovar: just *Psn* strains were clustered in a single SG, while *Psf* and *Psv* strains were resolved in two and three SGs, respectively (Table 1).

**Figure 2:**
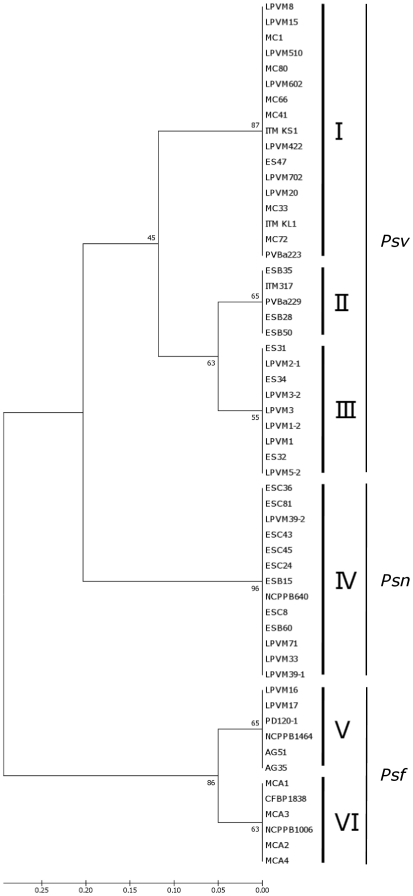
SNP group-based *P. savastanoi* phylogenetic analysis. UPGMA dendrogram constructed using the concatenated bases for the ten SNPs obtained for each of the 56 *P. savastanoi* strains. On the right of the dendrogram the designation of each cluster is reported, as pathovars (*Psv*, *Psn* and *Psf*) and as SGs (roman numbers). Bootstrap values (1,000 replicates) are indicated on the corresponding nodes. Genetic similarity among strains and pathovars was estimated by p-distance method, computed on MEGA 5.

The genetic distances among both *P. savastanoi* strains and the pathovars they belong to were also calculated and graphically reported. To this aim the ten-bases representing the SNP codes of all the strains tested were aligned using ClustalW2 software with default settings. The results obtained were used as an input file for MEGA5 software to produce an UPGMA dendrogram (Figure 2).

Three main branches were firstly obtained, each including all and only the strains belonging to one of the three *P. savastanoi* pathovars. A further intra-pathovar clustering was generated, strongly supported by bootstrap analysis. As expected, *Psv*, *Psf*, and *Psn* strains grouped in three, two, and just one minor branches respectively, each corresponding to a single SG (Table 1).

The main finding of these results is that HRMA is not only an excellent technique in the interrogation of SNPs derived from sequencing approaches, but also a powerful and low-cost technique for the discovery of unknown SNPs, highly informative for bacterial genotyping, as demonstrated by the unexpected SNP variants unveiled here by HRMA.

### One step HRM-multiplex assay for *P. savastanoi* pathovars discrimination

With the aim of setting up a HRM-multiplex assay able to discriminate in one step the three *P. savastanoi* pathovars, the primer pairs for the SNP markers able to group together all the *Psn* strains (C2 and R) were coupled with those able to group *Psf* strains (L1, L2 and J). Six different combinations were firstly tested on DNA template from the representative strains *Psv5*, *Psn23* and *Psf134*.

**Figure 3:**
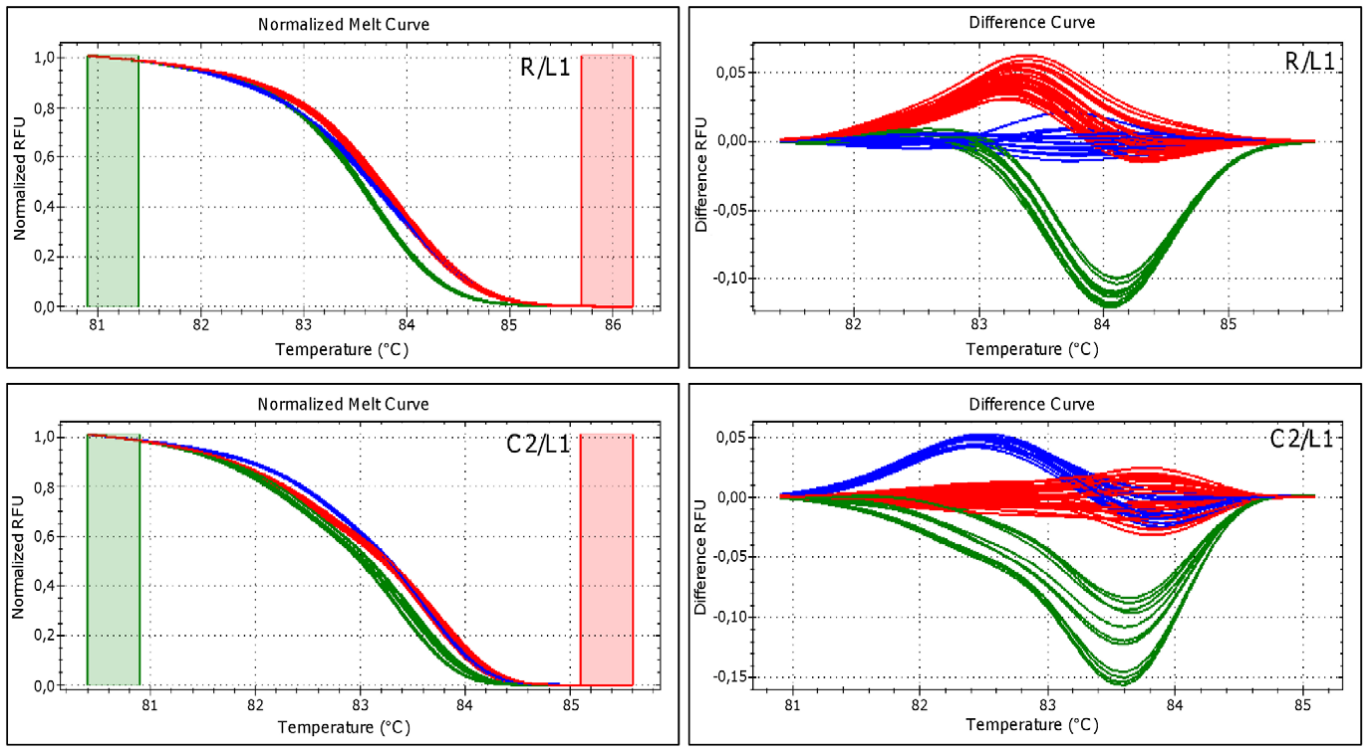
Discrimination of *P. savastanoi* pathovars by multiplex HRM analysis. Normalized and difference plots obtained in multiplex HRM assays, performed using the two couples of primer pairs for SNP markers R/L1 and C2/L1 on the 56 *P. savastanoi* strains examined here. On each plot different colors indicate distinct profiles, having a pathovar-specific distribution (*Psv*, *Psn* and *Psf* strains, giving red, blue and green traces, respectively). RFU: Relative fluorescence units. Green and red columns represent pre- and post-melting normalization regions.

The results obtained are shown in Figure S3. The combined use of the six primer pairs was able to clearly discriminate the three *P. savastanoi* pathovars, as demonstrated by their normalized melting curves. The couples of primer pairs for SNP markers C2/L2, C2/J, R/J, and R/L2 produced double peaked derivative melting curves. The two peaks were clearly originated from the melting of two amplicons with different melting temperatures. On the contrary, the couples of primer pairs for the SNP markers R/L1 and C2/L1 gave a single peak. Two amplicons were produced in these cases as well, but differing less than 1°C in their melting temperatures. Since multiple peak curves represent a complex pattern that may be difficult to be interpreted, further analysis were carried out only with the primers for the couples of SNPs C2/L1 and R/L1, using the 56 *P. savastanoi* strains reported in Table 1. In Figure 3 HRM normalized and difference plots for each multiplex assay are shown, representing the results obtained by using 10 ng of pure genomic DNA for reaction. The three *P. savastanoi* pathovars could be consistently resolved in three clearly different curves, using all the 56 strains tested in this work, and with both the couples of primer pairs used. The same results (99.0 percent of confidence) were also obtained using ten-fold diluted DNA template from thermal lysis. If undiluted thermal lysed DNA was used, “temperature shifted” option (HRM analysis software, Bio-Rad Laboratories, Inc.) was applied to impose a further normalization point (data not shown). The sensitivity of this multiplex HRMA-based protocol was evaluated by testing different amounts of genomic DNA (from 10 ng to 1 fg per reaction). In Figure S4 the LLOD for the couples of primer pairs for the SNP markers R/L1 and C2/L1 are shown, obtained using DNA from strains *Psv5*, *Psn23* and *Psf134*. Detection thresholds of 1 pg and 10 fg of DNA template were found for markers R/L1 and C2/L1, respectively. With these DNA amounts was still possible to gain distinct melt curve profiles for each of the three *P. savastanoi* pathovars in HRM analysis.

The primers for the SNP markers C2/L1, showing the lowest LLOD, were further tested for their ability to specifically discriminate the three *P. savastanoi* pathovars examined here from several phylogenetically related bacteria or reported to be ubiquitously associated to plants, water and soil (Table S1). To this purpose pure DNAs (10 fg/reaction) extracted from these bacteria were used as template and the results are reported in Figure S5. Each bacterial species or pathovar clustered separately and distinct HRM normalized curves were obtained, with the exception of the strains of *P. alcaliphila* and *P. fluorescens* studied here, that were unable to give HRM-analyzable amplifications. Importantly, unique, characterizing and reproducible profiles were gained for *Psv*, *Psn* and *Psf*, each of them clustering independently from the other bacteria. These data unequivocally demonstrated the high discriminatory power of the one step HRM-multiplex assay developed here for *Psv*, *Psn* and *Psf*, also able to specifically distinguish each of these pathovars from other related bacteria.

### Application of HRM analysis to *P. savastanoi* infected Oleander plants

The couple of primer pairs for SNPs C2/L1 was further analyzed for its potential application as an *in vivo* diagnostic tool to detect *P. savastanoi* on and into infected plants. To this purpose a global approach was firstly adopted to simultaneously evaluate any possible *in planta* cross-reaction of these primers with both culturable and unculturable bacteria naturally resident on Oleander plants, and the presence of any plant compound having PCR inhibitory activity. Detached Oleander leaves were artificially and separately inoculated, on the upper or on the lower surface, with *Psv5*, *Psn23* or *Psf134* bacterial suspensions, as described in Materials and Methods section. Thermal lysed DNA obtained from leaf washings was used as template in HRM Real-Time PCR with the couple of primer pairs for SNPs C2/L1. Pure genomic DNAs of *Psv5*, *Psn23* and *Psf134* were used as positive controls. The results obtained with 5 and 50 µl bacterial drop volumes are shown in Figure S6, with two replicates for each leaf side and the three positive controls. No aspecific signals attributable to the natural epiphytic microflora were ever obtained, nor was any interference found in the ability of this HRM protocol to specifically detect and discriminate the three *P. savastanoi* pathovars in Oleander leaf washings. Similar HRM plots and pathovar-specific clustering were also produced using bacterial drop volumes of 10, 20 and 100 µl (data not shown). Moreover, information on the culturable and unculturable bacterial epiphytes present on these Oleander leaf samples were gained. A 16S rDNA library of 384 clones was obtained using as template thermal lysed DNA from leaf washings of negative controls, as described in Materials and Methods section. Each clone was then subjected to ARDRA using *AluI* enzyme [61], and a total of 81 different ARDRA profiles were observed (data not shown). Sequencing of 16S rDNA was carried out on two clones for each ARDRA profile. In Table S2 the results obtained by BLAST analysis [64] are shown, having a degree of similarity with their closest matches in the range of 97–99%. About 23% of ARDRA profiles were derived from sequences giving closest matches with genus *Pseudomonas*, while clones related to bacteria from genera *Xantomonas* (15%), *Bacillus* (14%), *Erwinia* (11%), *Acinetobacter* (9%) and *Lactobacillus* (2%) were represented to a lesser extent. Unsurprisingly more than a quarter of the sequences giving the 81 different ARDRA profiles were found highly homologous to several clones from 16S rRNA genes of unculturable bacteria. These data further supported the strong discriminative power of primer pairs for SNPs C2/L1 for the specific detection of *Psv*, *Psn* and *Psf* against a wide range of bacterial genera commonly associated to plants as epiphytes.

**Figure 4:**
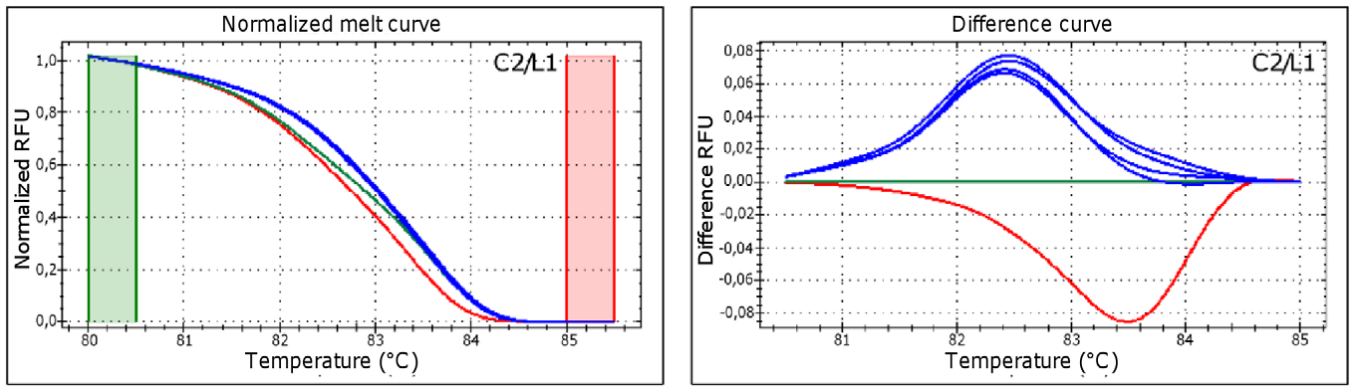
*In planta* performances of multiplex HRM analysis for discrimination of *P. savastanoi* pathovars. Normalized and difference plots obtained in multiplex HRM assay, performed using the couple of primer pairs for SNP markers C2/L1 on Oleander plants inoculated with *Psn23*. Pure genomic DNAs from *Psv5*, *Psn23*, and *Psf134* were also tested as controls. On each plot different colors indicate distinct profiles, generated by *Psv5*, *Psn23* and *Psf134* (red, blue and green trace, respectively). Three *Psn23* artificially infected plants were tested for each of the three independent Real-Time PCR runs performed. RFU: Relative fluorescence units. Green and red columns represent pre- and post-melting normalization regions.

The same primers combination was then used to detect *Psn* strains in artificially infected *in vitro* Oleander plants. Figure 4 shows normalized and difference HRM plots obtained with DNA template extracted from three different *Psn*-infected plants, compared with those produced by pure DNA from *Psv5*, *Psn23* and *Psf134* strains. No variation was ever observed in the performances of this HRMA-based assay when using as template DNA extracted from *Psn*-infected plants, in comparison with *Psn23* pure DNA, confirming the *in vivo* pathovar-specificity of our multiplex HRM protocol as well. Identical plots were observed with all the *Psn* strains tested in this study (data not shown).

## Discussion

The evolution of HRMA from conventional melting curves analysis is based on the development of a novel type of dye, having a higher dsDNA saturating activity than SYBR Green I, called EvaGreen®, and of a new generation of thermal cyclers, finely tuning temperature increments/decrements up to 0.1°C [72]–[73]. Since then, HRMA is emerging as a fundamental technique in any field of research where the rapid and sensitive detection of a single nucleotide mutation was so strongly informative to genotype and categorize closely related species or cellular types. In the last few years an increasing number of papers have been published on HRMA specifically applied to the identification of human and animal pathogens [47], [50], [74]–[76], to the genotyping of drug-resistant bacterial isolates [77]–[79], or to human genetic variants linked to cancer [45]–[46], where an earlier diagnosis is essential to gain important clinical benefits.

Here for the first time we reported the successful use of HRMA on a bacterial plant pathogen, *P. savastanoi*, the causal agent of Olive and Oleander knot disease.

A total number of 56 strains, belonging to the three pathovars *Psv*, *Psn,* and *Psf* and having different geographical origins, were tested using a SNPs panel, designed according to nine loci of the TTSS cluster sequences of the three representative strains *Psv5*, *Psn23* and *Psf134.* In particular, nine out of the ten SNPs interrogated here derived from the alignment of TTSS sequences of strains *Psv5*, *Psn23* and *Psf134.* On the contrary one of these SNPs was discovered as a result of HRMA application on the other 53 *P. savastanoi* strains, whose TTSS sequences were unknown and thus tested as blind samples.

Five of these SNPs generated unique HRM profiles able to group together and exclusively all the strains belonging to the same *P. savastanoi* pathovar. However, it was never possible to simultaneously discriminate the three pathovars using a single SNP marker. This goal was achieved by developing a multiplex HRM protocol using the primers for the couples of markers C2/L1 or R/L1: for each combination of primer pairs the pathovar identification carried out on the 56 *P. savastanoi* strains tested here was 100% coherent with the results obtained using the highly specific Real-Time PCR assay previously set up for this bacterium [41]. This test was also shown to be highly reproducible in independent experiments as well as considerably sensitive, since a LLOD of 10 fg or 1 pg of DNA template was obtained for markers C2/L1 and R/L1, respectively. This assay is also extremely rapid since it produced the expected results in less than 90 minutes with no need of further procedures, being HRMA a closed-tube and one-step method. Furthermore, among the few multiplex HRMA assays developed so far [58]–[59] this the first test capable of overcoming an important weak point of HRMA-multiplexing, that is to type variants related to several different amplicons at the same time producing single melting discriminative curves instead of a complex pattern, as usually occurs in these cases.

The primer pairs for SNPs C2/L1, having the lowest LLOD, were further analyzed to evaluate the potential applicability of the multiplex HRMA protocol developed here as an *in vivo* diagnostic tool for the detection of these *P. savastanoi* pathovars. Firstly, these primers were demonstrated to be able to specifically discriminate pure DNAs of *Psv, Psn* and *Psf* from those of several other bacteria, phylogenetically related to *P. savastanoi* or ubiquitous. Moreover the pathovar-specificity of this HRMA-based assay was demonstrated not to be lowered or altered by any microbial contaminant DNA that may be present on or into *P. savastanoi* host plants, as assessed in preliminary experiments carried out on micropropagated and adult Oleander plants, artificially and individually inoculated with *Psv5*, *Psn23* and *Psf134* strains. These data clearly demonstrated the power of our assay to overcome also one of the main problems that could be faced with HRMA, that is its limit in resolving polymicrobial infections or consortia. To this concern, the epiphytic bacteria resident on the Oleander leaf samples examined here were investigated, and the data obtained were in accordance with previous findings on *P. savastanoi* host plants [27], [80]. Unaltered performances were obtained also when this HRMA assay was carried out on DNA template from *Psv5*, *Psn23* and *Psf134* spiked with total DNAs extracted from Olive, Oleander and Ash leaf washings (data not shown).

The detection of genetic variations phylogenetically informative is also pivotal for the analysis of the evolutionary history of a pathogen and of its populations, in order to increase the knowledge about its epidemiology. Among the methods currently available, multi-locus sequence typing (MLST) and SNP typing are those having the highest degree of specificity, sensitivity, and reproducibility, relying on the direct comparison of specific nucleotide sequences. The diversity among bacteria belonging to the *P. syringae* complex was explored by MLST carried out on several housekeeping genes/loci of the so called “core genome”, to assess the role of recombination events in the evolution of *P. syringae* related pathogens. A high level of clonality was observed within these species and five major phylogroups were identified [81]. Very recently similar conclusions were reached by analyzing the single gene *rpoD* [82] or by a microarray approach [83]. However, when MLST was performed on a wide collection of *P. syringae* strains, belonging to the pathovars *tomato*, *maculicola*, *apii*, *antirrhini*, and *avellanae*, recombination was also found to strongly contribute to bacterial diversity [84]–[85]. Actually, this could be an expected result when examining closely related strains, which have more opportunities to recombine than distantly related bacteria, because of their DNA homology, overlapping host ranges, and ecological distribution. Moreover, *P. syringae* strains causing new diseases could be properly allocated into genomospecies and pathovars by MLST [86]–[87].

As far as *P. savastanoi* is concerned, no comprehensive MLST phylogeny studies were carried out so far, including the three pathovars examined here. Up to now only an extensive analysis by fAFLP was developed for these *P. savastanoi* pathovars [15].

The HRMA-based SNP typing method developed here was also demonstrated to have a high discriminatory power in resolving *P. savastanoi* pathovars and strains, and provided results largely coherent with those previously obtained by fAFLP analysis and even more detailed [15]. Moreover, our findings support the most recent classification of these pathovars within the *P. savastanoi* species, based on laborious and skilled DNA/DNA hybridization experiments [2]. This HRM-typing protocol was developed according to the SNP variants found on *P. savastanoi hrp* genes. Our preliminary data suggest it could be the ideal instrument to fully determine whether, and to which extent, the evolution of TTSS contributed to shape the differentiation of *P. syringae* complex, including *P. savastanoi* pathovars. To this concern, *hrp* genes were postulated to have been introduced early and by horizontal gene transfer (HGT) into a *P. syringae* ancestor from a still unknown enteric animal pathogen, without further significant HGT events driving the evolution of TTSS in these bacteria [88]. Thus it was supposed that *hrp* genes likely followed the same evolutionary course of the whole genome although at a faster rate, as assessed for a broad range of genes coding for virulence associated factors that were demonstrated to evolve considerably in non-host settings as well [89]–[90]. However, in several *P. syringae* species a positive selection was demonstrated to act on mutations occurring on *hrpA*, aimed at maintaining diversity at this locus that was strongly functional in different host contexts [90].

If the phylogenetic discriminative potential of this HRMA-typing protocol was confirmed by examining a larger number of *P. savastanoi* strains and other *P. syringae* species, this method would provide a more rapid and economic tool to identify the evolutionary relationships among bacteria belonging to the *P. syringae* complex, in comparison to other more expensive sequence typing approaches used so far [81], [86]–[87].

HRMA is a low-cost identification method compared to other diagnostic assays because no labeled expensive probes or specialized reagents are required, but just generic PCR primers and a high saturating dsDNA dye are needed. Hence, the pathovar-specific HRMA-based protocol for *P. savastanoi* developed here, working both *in vitro* and *in planta* to easily discriminate and detect *Psv*, *Psn* and *Psf* strains, can facilitate and promote high-throughput screenings aimed at revealing important epidemiological data and trends of this species, such as host range, temporal or geographical tendencies and other fundamental observations related to pathogenicity/virulence. Besides the intrinsic high relevance of these epidemiogical findings, these data would also strongly affect the management of the diseases that *P. savastanoi* causes, helping to correctly calibrate preventive disease control measures concerning this phytopathogen. Since HRMA can be performed in a rapid, economical and convenient way, it could be easily applied in routine identification of *P. savastanoi* pathovars in plant propagation materials, by a simple high-throughput process.

## Supporting Information

Figure S1 **Alignment of TTSS cluster sequences of** ***Psv5, Psn23*** **and** ***Psf134*** **, location of SNPs and primers.** Annealing sites for the nine primers pairs are underlined. Primer directions are indicated by black arrows. SNPs are highlighted in red and the SNP marker name is reported in capital letter. Position of SNP marker JL1 is highlighted in yellow.
(PDF)

Figure S2 **Difference HRM plots for all the SNPs screened in the study.** Difference HRM plots of the amplicons obtained from the 56 *P. savastanoi* strains using the nine primer pairs. For each amplicon, the corresponding SNP marker is reported on the upper-right corner of the plot. Different colors are used to indicate distinct profiles, corresponding to the HRMA-based clustering of the 56 *P. savastanoi* strains into pathovars or SGs. RFU: Relative fluorescence units.
(TIFF)

Figure S3 S**etting up of multiplex HRMA assay.** Normalized plots and derivative melting peaks obtained in multiplex HRM assay design, using six duplex combinations of the primer pairs referred to the SNP markers reported on each plot. Pure genomic DNAs (10 ng) of representative strains *Psv5, Psn23* and *Psf134* (red, green and blue traces, respectively) were separately used as templates. RFU: Relative fluorescence units. Green and red columns represent pre- and post-melting normalization regions.
(TIF)

Figure S4 **Evaluation of Lower Limit of Detection (LLOD) of the multiplex HRMA assay.** Normalized and difference HRM plots obtained with the combinations of primer pairs for SNP markers R/L1 (upper plots) and C2/L1 (lower plots), using the lowest detectable quantity of DNA template (1 pg and 10 fg, respectively). On each plot red, green and blue traces were produced by *Psv5, Psn23* and *Psf134*, respectively. RFU: Relative fluorescence units. Green and red columns represent pre- and post-melting normalization regions.
(TIF)

Figure S5 **HRMA multiplex assay on bacteria related to** ***P. savastanoi*** **.** (A) Normalized melting curves obtained by using as template pure DNAs (10 pg/reaction) extracted from bacteria closely related to *P. savastanoi* (Table S1), and the primer pairs for SNP markers C2/L1. Pure DNAs from *Psv5*, *Psn23* and *Psf134* were used as positive controls. Traces of different color indicate distinct HRMA profiles, produced by these bacteria and according to the color code reported in Table S1. No HRMA detectable signal was obtained for *P. alcaliphila* str. 28. and *P. fluorescens* str. 11. (B) Magnification of black-squared area in (A).
(TIFF)

Figure S6 **Specificity of multiplex HRM assay for** ***P. savastanoi*** **pathovars detection tested against Oleander epiphytes.** Normalized and difference HRM plots obtained by multiplex HRM assay performed with the combination of primer pairs for SNP markers C2/L1. Washings from Oleander leaves artificially surface-inoculated with *Psv5, Psn23* and *Psf134* were used for thermal lysis extraction of DNA, to be used as template. Bacterial suspension drops of 5 µl (upper plots) and 50 µl (lower plots) were spotted on each leaf side. Pure genomic DNAs from *Psv5, Psn23* and *Psf134* were also tested as controls. On each plot red, green and blue traces were produced by *Psv5, Psn23* and *Psf134*, respectively. RFU: Relative fluorescence units. Green and red columns represent pre- and post-melting normalization regions.
(TIF)

Table S1 **Bacteria related to** ***P. savastanoi*** **or ubiquitous used in this study.**
(DOC)

Table S2 **Identification of epiphytic bacterial genera present on Oleander leaf washings using 16S rDNA gene sequencing, according to BLAST analysis.**
(DOC)
