# Supplementary material for: High-Resolution Melting Analysis as a Powerful Tool to Discriminate and Genotype Pseudomonas savastanoi Pathovars and Strains
Source: PLoS One. 2012 Jan 25;7(1):e30199. doi: 10.1371/journal.pone.0030199 (PMC3266268; doi:10.1371/journal.pone.0030199)
Supplement: Figure S1 — Alignment of TTSS cluster sequences of Psv5, Psn23 and Psf134 , location of SNPs and primers. Annealing sites for the nine primers pairs are underlined. Primer directions are indicated by black arrows. SNPs are highlighted in red and the SNP marker name is reported in capital letter. Position of SNP marker JL1 is highlighted in yellow. (PDF) [file pone.0030199.s001.pdf]

```
Psv5          ATGAGCACAGACATTGATAAGGGCGTCCGAGAGTATTGGGACGTAACTGCATTATCCGCT 60
Psn23         ATGAGCACAGACATTGATAAGGGCGTCCGAGAGTATTGGGACGTAACTGCATTATCCGCT 60
Psf134        ATGAGCACAGACATTGATAAGGGCGTCCGAGAGTATTGGGACGTAACTGCATTATCCGCT 60
              ************************************************************

Psv5      \\  TGTTTGGAGTGGTCAGCGGTGCGTACACCGGTGCTGACCGCTCCAGAGTCGGCTACATCG 1260
Psn23         TGTTTGGAGTGGTCAGCGGTGCGTACACCGGTGCTGACCGCTCCAGAGTCGGCTACATCG 1260
Psf134        TGTTTGGAGTGGTCAGCGGTGCGTACACCGGTGCTGACCGCTCCAGAGTCGGCTACATCG 1260
              ************************************************************

                 hrpS_for
               ------------->
Psv5          AAGCGGCACAAGGCGGAACGCTGTATCTGGATGAGATCGACAGCATGCCGCTCAGCCTGC 1320
Psn23         AAGCGGCACAAGGCGGAACGCTGTATCTGGATGAGATCGACAGCATGCCGCTCAGCCTGC 1320
Psf134        AAGCGGCACAAGGCGGAACGCTGTATCTGGATGAGATCGACAGCATGCCGCTCAGCCTGC 1320
              ************************************************************

Psv5          AGGCCAAGCTGCTGAGGGTGCTGGAAACCCGAGCACTGGAACGACTGGGTTCGACATCGA 1380
Psn23         AGGCCAAGCTGCTGAGGGTGCTGGAAACCCGAGCACTGGAACGACTGGGTTCGACATCGA 1380
Psf134        AGGCCAAGCTGCTGAGGGTGCTGGAAACCCGAGCACTGGAACGACTGGGTTCGACATCGA 1380
              ************************************************************

              S                            hrpS_rev
              |                      <--------------
Psv5          TGATCAAGCTGGATGTCTGCGTGATCGCTTCGGCCCAGTCGTCTCTGGATGATGCCGTCG 1440
Psn23         CGATCAAGCTGGATGTCTGCGTGATCGCTTCGGCCCAGTCGTCTCTGGATGATGCCGTCG 1440
Psf134        CGATCAAGCTGGATGTCTGCGTGATCGCTTCGGCCCAGTCGTCTCTGGATGATGCCGTCG 1440
               ***********************************************************

Psv5          AGCAGGGGAAATTTCGTCGGGATCTGTACTTTCGCCTTAACGTGTTGACACTCCAGCTGC 1500
Psn23         AGCAGGGGAAATTTCGTCGGGATCTGTACTTTCGCCTTAACGTGTTGACACTCCAGCTGC 1500
Psf134        AGCAGGGGAAATTTCGTCGGGATCTGTACTTTCGCCTTAACGTGTTGACACTCCAGCTGC 1500
              ************************************************************

                                                hrpJ_for
                                             --------------->
Psv5      \\  TGAAAAGACCGGTGTCGTGGTTACGGTCGCAACCAGCGACATGAACCGCGCCGTACGTGT 4020
Psn23         TGAAAAGACCGGTGTCGTGGTTACGGTCGCAACCAGCGACATGAACCGCGCCGTACGTGT 4020
Psf134        TGAAAAGACCGGTGTCGTGGTTACGGTCGCAACCAGCGACATGAACCGCGCCGTACGTGT 4020
              ************************************************************

                                     J                              hrpJ_rev
                                     |                             <-------
Psv5          GCTTAACGCTGCCGGCCTGCCACGCCAGTCGCGCGCCAGTCTGGGCGACATCTTCAAGAA 4080
Psn23         GCTTAACGCTGCCGGCCTGCCACGCCAGTCGCGCGCCAGTCTGGGCGACATCTTCAAGAA 4080
Psf134        GCTTAACGCTGCCGGCCTGCCACACCAGTCGCGCGCCAGTCTGGGCGACATCTTCAAGAA 4080
              *********************** ************************************

Psv5          AGAAGGGGTCATCTCGACTCCTCTCGAAGAGCGTGCCCGCTACATCTATGCCCTGTCTCA 4140
Psn23         AGAAGGGGTCATCTCGACTCCTCTCGAAGAGCGTGCCCGCTACATCTATGCCCTGTCTCA 4140
Psf134        AGAAGGGGTCATCTCGACTCCTCTCGAAGAGCGTGCCCGCTACATCTATGCCCTGTCTCA 4140
              ************************************************************

Psv5          GGAGCTTGAAGCCACGTTGTCGCAGATAGACGGTGTCATCGTGGCCAGAGTGCATGTGGT 4200
Psn23         GGAGCTTGAAGCCACGTTGTCGCAGATAGACGGTGTCATCGTGGCCAGAGTGCATGTGGT 4200
Psf134        GGAGCTTGAAGCCACGTTGTCGCAGATAGACGGTGTCATCGTGGCCAGAGTGCATGTGGT 4200
              ************************************************************

Psv5      \\  GACACTCAAAGCCTGCCTCTGGATTCCAGCGGCATCGATACCGGCGCCTTGCAACAGGGC 7260
Psn23         GACACTCAAAGCCTGCCTCTGGATTCCAGCGGCATCGATACCGGCGCCTTGCAACAGGGC 7260
Psf134        GACACTCAAAGCCTGCCTCTGGATTCCAGCGGCATCGATACCGGCGCCTTGCAACAGGGC 7260
              ************************************************************
```

```
                hrpC1_for                            C1
                ------->                              |
Psv5     CTTGATCGGGTGTTGAGCTATGGCAGCGGCAGCAAGAAATCCGGGAAAAGCCGTTCCGGT 7320
Psn23    CTTGATCGGGTGTTGAGCTATGGCAGCGGCAGCAAGAAATCCGTGAAAAGCCGTTCCGGT 7320
Psf134   CTTGATCGGGTGTTGAGCTATGGCAGCGGCAGCAAGAAATCCGGGAAAAGCCGTTCCGGT 7320
         ******************************************* ****************

                              hrpC1_rev
                         <--------
Psv5     GGCCGCGCGAACATTCGTGTAACAGCCGATGTGCGCAACAACGCGGTACTGATTTATGAC 7380
Psn23    GGCCGCGCGAACATTCGTGTAACAGCCGATGTGCGCAACAACGCGGTACTGATTTATGAC 7380
Psf134   GGCCGCGCGAACATTCGTGTAACAGCCGATGTGCGCAACAACGCGGTACTGATTTATGAC 7380
         ************************************************************

Psv5     TTGCCGTCGCGCAAGCCGATGTACGAGAAACTCATCAAGGAACTGGACGTCTCACGCAAC 7440
Psn23    TTGCCGTCGCGCAAGCCGATGTACGAGAAACTCATCAAGGAACTGGACGTCTCACGCAAC 7440
Psf134   TTGCCGTCGCGCAAGCCGATGTACGAGAAACTCATCAAGGAACTGGACGTCTCACGCAAC 7440
         ************************************************************

Psv5   \\ GCCTGGCCTCACGCCTGGCTGCAACCGGGTGAAGAGAGCGAGGTGTATATCGCTGTGCGC 8520
Psn23  \\ GCCTGGCCTCACGCCTGGCTGCAACCGGGTGAAGAGAGCGAGGTGTATATCGCTGTGCGC 8520
Psf134 \\ GCCTGGCCTCACGCCTGGCTGCAACCGGGTGAAGAGAGCGAGGTGTATATCGCTGTGCGC 8520
         ************************************************************

               hrpC2_for                                        C2
               ------->                                          |
Psv5     CAGCCACAGATATCGAAAATGGCGAAAGAGAGTCGGCCATCACTGCTCAAGGGAGCGAAA 8580
Psn23    CAGCCACAGATATCGAAAATGGCGAAAGAGAGTCGGCCATCACTGCTCAAGGGAGCGCAA 8580
Psf134   CAGCCACAGATATCGAAAATGGCGAAAGAGAGTCGGCCATCACTGCTCAAGGGAGCGAAA 8580
         ********************************************************* **

                                   hrpC2_rev
                              <--------
Psv5     CCATGAAGATCAGTAGCATTGCAGTTGTGCTGGTGCTGTTCGCTACCCTGTCGGGGTGTG 8640
Psn23    CCATGAAGATCAGTAGCATTGCAGTTGTGCTGGTGCTGTTCGCTACCCTGTCGGGGTGTG 8640
Psf134   CCATGAAGATCAGTAGCATTGCAGTTGTGCTGGTGCTGTTCGCTACCCTGTCGGGGTGTG 8640
         ************************************************************

Psv5   \\ CAGTCGGCGCGAGCATCATGACTGGGGCATCGCGCTGCACATCGAAGGGCGTGCGCTGCG 8940
Psn23  \\ CAGTCGGCGCGAGCATCATGACTGGGGCATCGCGCTGCACATCGAAGGGCGTGCGCTGCG 8940
Psf134 \\ CAGTCGGCGCGAGCATCATGACTGGGGCATCGCGCTGCACATCGAAGGGCGTGCGCTGCG 8940
         ************************************************************

              hrpV_for
              -------->
Psv5     TCCCGAGCAACTGAGAGAGGGCGCTGCAAATGAGGTTCTCAGAGGCCGAGCGGTTCCGTAA 9000
Psn23    TCCCGAGCAACTGAGAGAGGGCGCTGCAAATGAGGTTCTCAGAGGCCGAGCGGTTCCGTAA 9000
Psf134   TCCCGAGCAACTGAGAGAGGGCGCTGCAAATGAGGTTCTCAGAGGCCGAGCGGTTCCGTAA 9000
         ************************************************************

             V
             |
Psv5     CTACTTTCTGTTTCTGGATGTACAGCGTGATTTTGTAGTGTGGCATGCCGTAAGCGACGC 9060
Psn23    CTACGTTCTGTTTCTGGATGTACAGCGTGATTTTGTAGTGTGGCATGCCGTAAGCGACGC 9060
Psf134   CTACTTTCTGTTTCTGGATGTACAGCGTGATTTTGTAGTGTGGCATGCCGTAAGCGACGC 9060
         **** *******************************************************

                                  hrpV_rev
                           <--------
Psv5     GCCGGATGCCGTCACCAACCTGGATGACATACGGCGACATGAGTTGATGCTGGCAGGCCT 9120
Psn23    GCCGGATGCCGTCACCAACCTGGATGACATACGGCGACATGAGTTGATGCTGGCAGGCCT 9120
Psf134   GCCGGATGCCGTCACCAACCTGGATGACATACGGCGACATGAGTTGATGCTGGCAGGCCT 9120
         ************************************************************
```

```
Psv5        GGAACACCTGGCTTAGGCAATTCTGCCAGCATGAAAGAAGACCGCCCCTGCAGAGGAGGC 9180
Psn23       GGAACACCTGGCTTAGGCAATTCTGCCAGCATGAAAGAAGACCGCCCCTGCAGAGGAGGC 9180
Psf134      GGAACACCTGGCTTAGGCAATTCTGCCAGCATGAAAGAAGACCGCCCCTGCAGAGGAGGC 9180
            ************************************************************

Psv5     \\ ATGCGCCACCGGGGCCATGACGAACATGGTTGCGGCCAGGGCGATGCCGTACAGGGCCAT 12840
Psn23    \\ ATGCGCCACCGGGGCCATGACGAACATGGTTGCGGCCAGGGCGATGCCGTACAGGGCCAT 12840
Psf134   \\ ATGCGCCACCGGGGCCATGACGAACATGGTTGCGGCCAGGGCGATGCCGTACAGGGCCAT 12840
            ************************************************************

                      hrpR_for
                      ------->
Psv5        GTTCGGCGGCACCTGCTGAACGCCAATTGCGTTGCGGGTGATCAGCAGGGTCATGGCGAT 12900
Psn23       GTTCGGCGGCACCTGCTGAACGCCAATTGCGTTGCGGGTGATCAGCAGGGTCATGGCGAT 12900
Psf134      GTTCGGCGGCACCTGCTGAACGCCAATTGCGTTGCGGGTGATCAGCAGGGTCATGGCGAT 12900
            ************************************************************

                                         R                hrpR_rev
                                         |               <--------
Psv5        TTTCAGAAAGGCGGTGCAGACAATCAACAGAAAGGGGATCAGTGACAGCGAGCCGAGAAA 12960
Psn23       TTTCAGAAAGGCGGTGCAGACAATCAACATAAAGGGGATCAGTGACAGCGAGCCGAGAAA 12960
Psf134      TTTCAGAAAGGCGGTGCAGACAATCAACAGAAAGGGGATCAGTGACAGCGAGCCGAGAAA 12960
            ***************************** ******************************

Psv5        CAGCGCCAGCATGATCGGGTTTACGCCCTCCATGATCATGATCGGGTCACCAGGCGGGTA 13020
Psn23       CAGCGCCAGCATGATCGGGTTTACGCCCTCCATGATCATGATCGGGTCACCAGGCGGGTA 13020
Psf134      CAGCGCCAGCATGATCGGGTTTACGCCCTCCATGATCATGATCGGGTCACCAGGCGGGTA 13020
            ************************************************************

Psv5     \\ ATCGCTCGCGCAGCGGGCGTGACGGCGCGAATCGGCGTGACCGTGTGGGTTGGCAATATA 20640
Psn23    \\ ATCGCTCGCGCAGCGGGCGTGACGGCGCGAATCGGCGTGACCGTGTGGGTTGGCAATATA 20640
Psf134   \\ ATCGCTCGCGCAGCGGGCGTGACGGCGCGAATCGGCGTGACCGTGTGGGTTGGCAATATA 20640
            ************************************************************

                                         ncJL_for
                                   ------------->
Psv5        GGCGGGGCGACGATTTTCATAGGACGGTTCTGAGCCTGGTCATGACCGCTGAGTGGGGCC 20700
Psn23       GGCGGGGCGACGATTTTCATAGGACGGTTCTGAGCCTGGTCATGACCGCTGAGTGGGGCC 20700
Psf134      GGCGGGGCGACGATTTTCATAGGACGGTTCTGAGCCTGGTCATGACCGCTGAGTGGGGCC 20700
            ************************************************************

                                                     JL1    JL2     <
                                                      |      |
Psv5        TGGCCAGGATTCGGTTCCCTTGTAAAAGGGGCTGACGCGTTTGTGCCAAAAGCTGTAGTG 20760
Psn23       TGGCCAGGATTCGGTTCCCTTGTAAAAGGGGCTGACGCGTTTGTGCCAAAAGCCGTAGTG 20760
Psf134      TGGCCAGGATTCGGTTCCCTTGTAAAAGGGGCTGACGCGTTTGTGCCAAAAGCTGTAGTG 20760
            ***************************************************** ******

               ncJL_rev
            ---------------
Psv5        ATAAAAACGGCGTTGCGCAAAAAAATGTATTACAAAGAATTACAAATTTTAAAATAATTA 20820
Psn23       ATAAAAACGGCGTTGCGCAAAAAAATGTATTACAAAGAATTACAAATTTTAAAATAATTA 20820
Psf134      ATAAAAACGGCGTTGCGCAAAAAAATGTATTACAAAGAATTACAAATTTTAAAATAATTA 20820
            ************************************************************

Psv5        TATAAATCAGTAACTTAAATAATTATTTTGGCTGGCACGGTTATCGCTATAGGGCTTGCA 20880
Psn23       TATAAATCAGTAACTTAAATAATTATTTTGGCTGGCACGGTTATCGCTATAGGGCTTGCA 20880
Psf134      TATAAATCAGTAACTTAAATAATTATTTTGGCTGGCACGGTTATCGCTATAGGGCTTGCA 20880
            ************************************************************

Psv5        CACCATTAAATTAAGGTAAGCCCATGTTTCCGAACCTAGTGATCCTTGATGCAACCCAGC 20940
Psn23       CACCATTAAATTAAGGTAAGCCCATGTTTCCGAACCTAGTGATCCTTGATGCAACCCAGC 20940
Psf134      CACCATTAAATTAAGGTAAGCCCATGTTTCCGAACCTAGTGATCCTTGATGCAACCCAGC 20940
            ************************************************************
```

```
                                                              hrpL1_for
Psv5      CACGCCAGGACTCTTCGTCTGCCGGTATCCGTCAACTGACGGCTGATCAGATACAGATGC 21000
Psn23     CACGCCAGGACTCTTCGTCTGCCGGTATCCGTCAACTGACGGCTGATCAGATACAGATGC 21000
Psf134    CACGCCAGGACTCTTCGTCTGCCGGTATCCGTCAACTGACGGCTGATCAGATACAGATGC 21000
          ************************************************************

                         L1
                         |
Psv5      TCAGAGCGTTCATTCAGAAGCGCGTAATGAACCCGGATGATGTCGATGACATCTTGCAAT 21060
Psn23     TCAGAGCGTTCATTCAGAAGCGCGTAATGAACCCGGATGATGTCGATGACATCTTGCAAT 21060
Psf134    TCAGAGCGTTCATTCGGAAGCGCGTAATGAACCCGGATGATGTCGATGACATCTTGCAAT 21060
          *************** ********************************************

              hrpL1_rev
Psv5      GCGTATTTCTGGAGGCCCTGCGCAACGAGCACAAGTTTCAACATGCCAGCAAGCCGCAGA 21120
Psn23     GCGTATTTCTGGAGGCCCTGCGCAACGAGCACAAGTTTCAACATGCCAGCAAGCCGCAGA 21120
Psf134    GCGTATTTCTGGAGGCCCTGCGCAACGAGCACAAGTTTCAACATGCCAGCAAGCCGCAGA 21120
          ************************************************************

          hrpL2_for
Psv5      CCTGGTTGTGTGGTATTGCGTTGAACCTGATCCGTAATCACTTCCGCAAAATGTATCGCC 21180
Psn23     CCTGGTTGTGTGGTATTGCGTTGAACCTGATCCGTAATCACTTCCGCAAAATGTATCGCC 21180
Psf134    CCTGGTTGTGTGGTATTGCGTTGAACCTGATCCGTAATCACTTCCGCAAAATGTATCGCC 21180
          ************************************************************

                                                    L2
                                                    |
Psv5      AGCCCTATCAGGAAAGCTGGGAAGACGACGTTCATTCAGGGCTGGAATGGAATGGCGATA 21240
Psn23     AGCCCTATCAGGAAAGCTGGGAAGACGACGTTCATTCAGGGCTGGAATGGAATGGCGATA 21240
Psf134    AGCCCTATCAGGAAAGCTGGGAAGACGACGTTCATTCAGAGCTGGAATGGAATGGCGATA 21240
          *************************************** ********************

                         hrpL2_rev
Psv5      TCACTCATCAGGTAGACGGGCACAGGCAATTGGCACGCGTCATCGCAGCCATTGATTGCT 21300
Psn23     TCACTCATCAGGTAGACGGGCACAGGCAATTGGCACGCGTCATCGCAGCCATTGATTGCT 21300
Psf134    TCACTCATCAGGTAGACGGGCACAGGCAATTGGCACGCGTCATCGCAGCCATTGATTGCT 21300
          ************************************************************

Psv5    //  AAGCACAAGGCGCAGAAGGCGTTCGATCATAACGTTGACCCGGTGCTGGATCAGTTCGGG 23820
Psn23   //  AAGCACAAGGCGCAGAAGGCGTTCGATCATAACGTTGACCCGGTGCTGGATCAGTTCGGG 23820
Psf134  //  AAGCACAAGGCGCAGAAGGCGTTCGATCATAACGTTGACCCGGTGCTGGATCAGTTCGGG 23820
            ************************************************************

Psv5      ATTGACCGGGCGCATTGA 23838
Psn23     ATTGACCGGGCGCATTGA 23838
Psf134    ATTGACCGGGCGCATTGA 23838
          ******************
```
