# Supplementary figures and images for: High-Resolution Melting Analysis as a Powerful Tool to Discriminate and Genotype Pseudomonas savastanoi Pathovars and Strains
Source: PLoS One. 2012 Jan 25;7(1):e30199. doi: 10.1371/journal.pone.0030199 (PMC3266268; doi:10.1371/journal.pone.0030199)

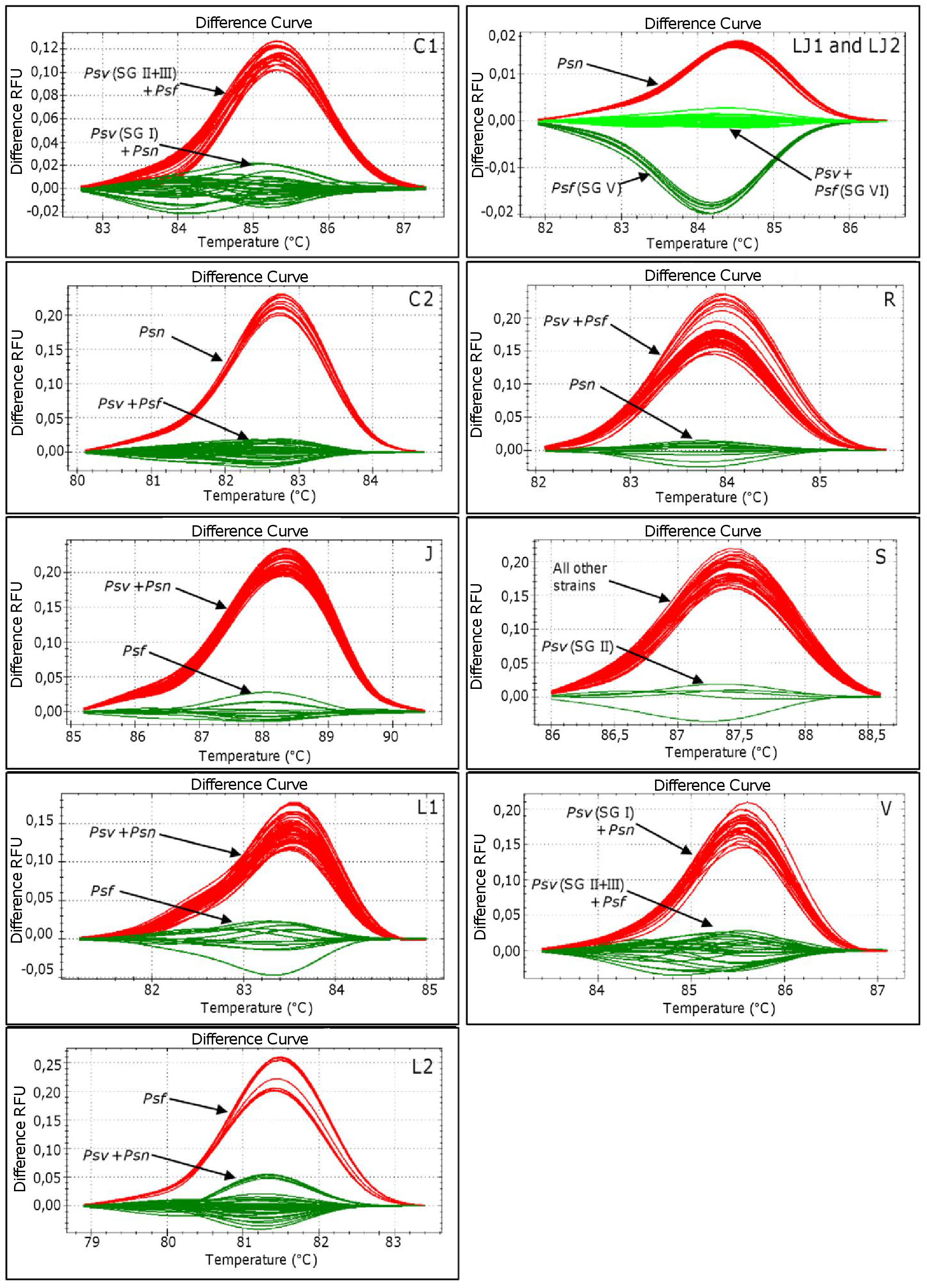

Supplement: Figure S2 — Difference HRM plots for all the SNPs screened in the study. Difference HRM plots of the amplicons obtained from the 56 P. savastanoi strains using the nine primer pairs. For each amplicon, the corresponding SNP marker is reported on the upper-right corner of the plot. Different colors are used to indicate distinct profiles, corresponding to the HRMA-based clustering of the 56 P. savastanoi strains into pathovars or SGs. RFU: Relative fluorescence units. (TIFF) [file pone.0030199.s002.tif]

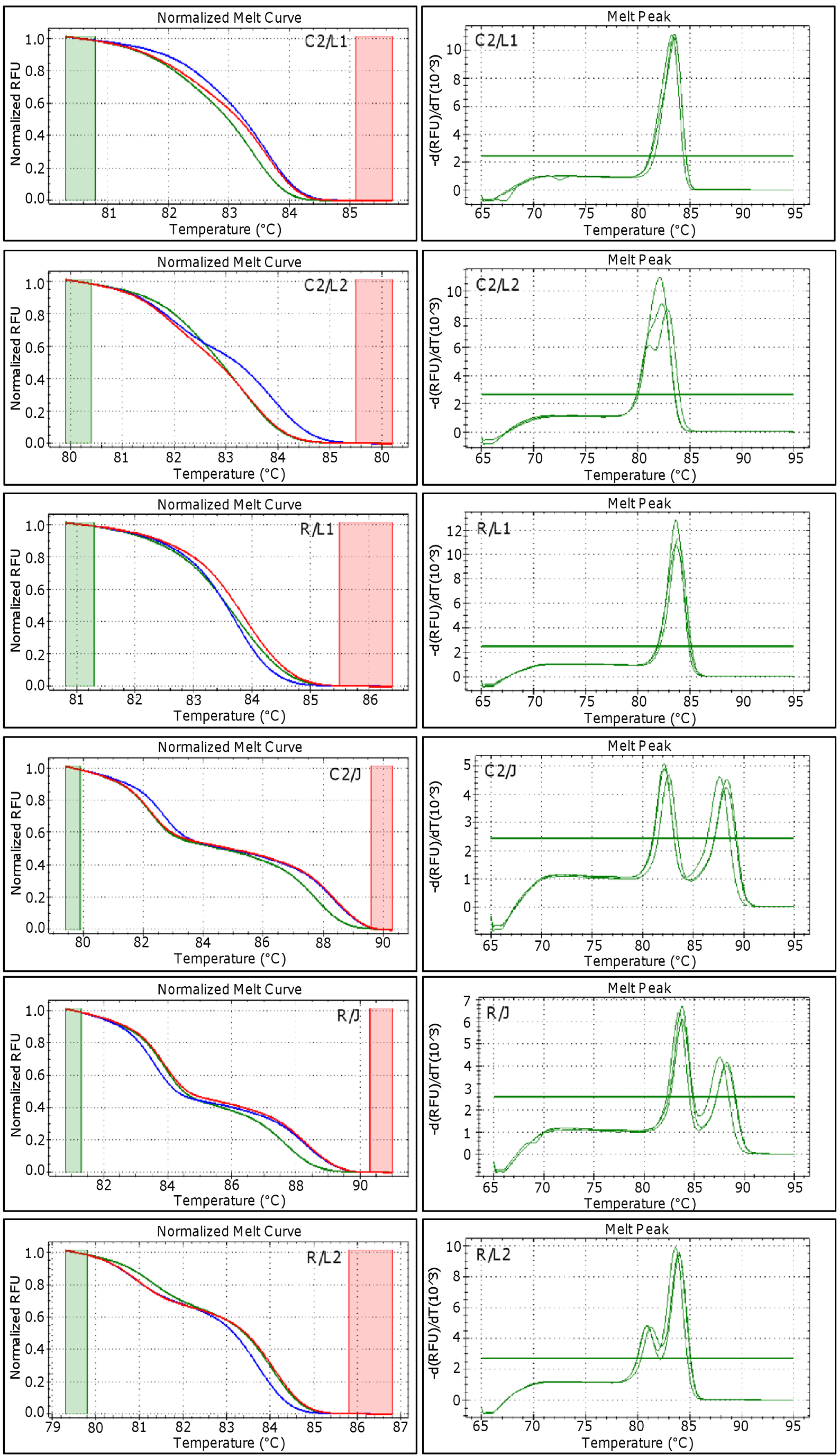

Supplement: Figure S3 — Setting up of multiplex HRMA assay. Normalized plots and derivative melting peaks obtained in multiplex HRM assay design, using six duplex combinations of the primer pairs referred to the SNP markers reported on each plot. Pure genomic DNAs (10 ng) of representative strains Psv5, Psn23 and Psf134 (red, green and blue traces, respectively) were separately used as templates. RFU: Relative fluorescence units. Green and red columns represent pre- and post-melting normalization regions. (TIF) [file pone.0030199.s003.tif]

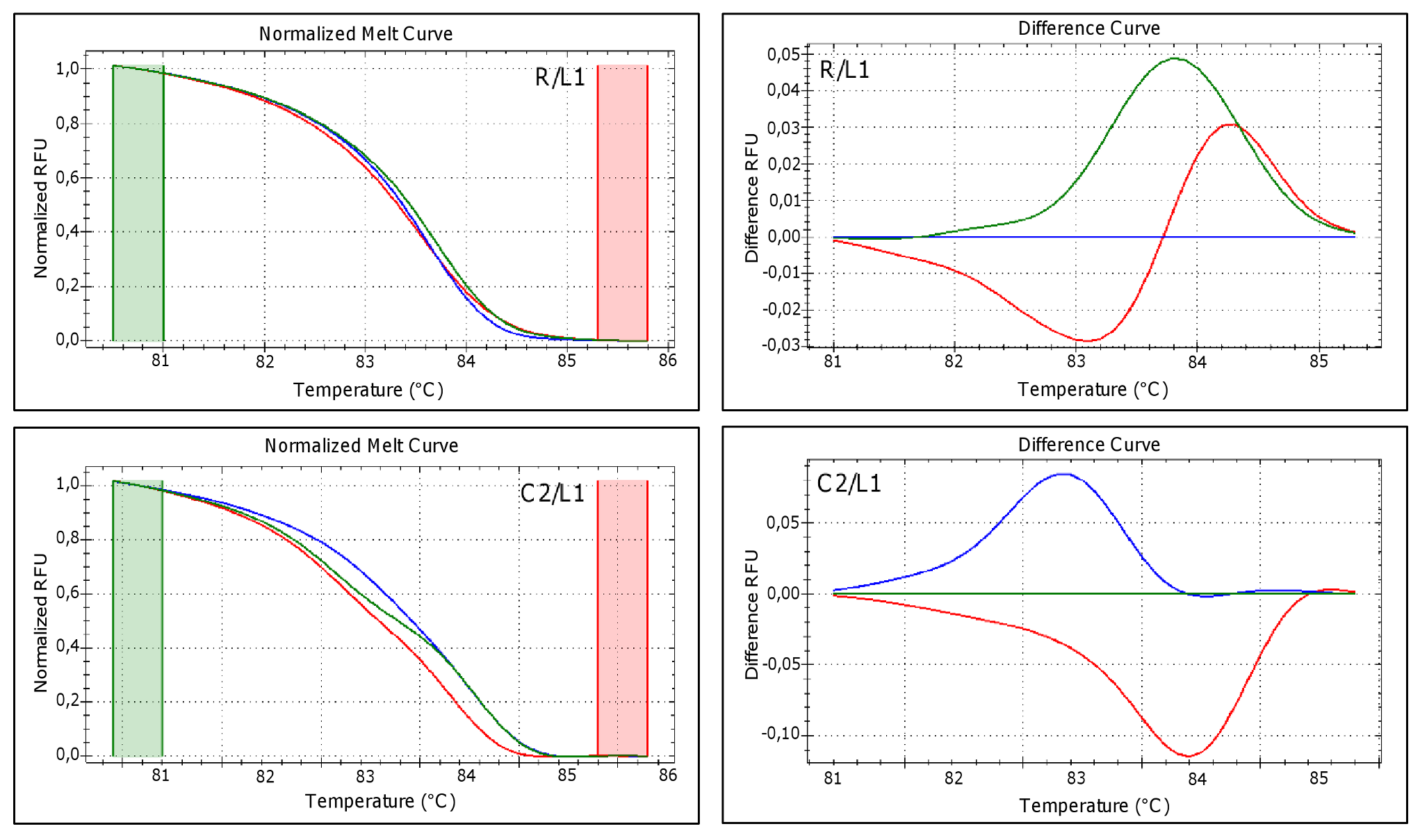

Supplement: Figure S4 — Evaluation of Lower Limit of Detection (LLOD) of the multiplex HRMA assay. Normalized and difference HRM plots obtained with the combinations of primer pairs for SNP markers R/L1 (upper plots) and C2/L1 (lower plots), using the lowest detectable quantity of DNA template (1 pg and 10 fg, respectively). On each plot red, green and blue traces were produced by Psv5, Psn23 and Psf134, respectively. RFU: Relative fluorescence units. Green and red columns represent pre- and post-melting normalization regions. (TIF) [file pone.0030199.s004.tif]

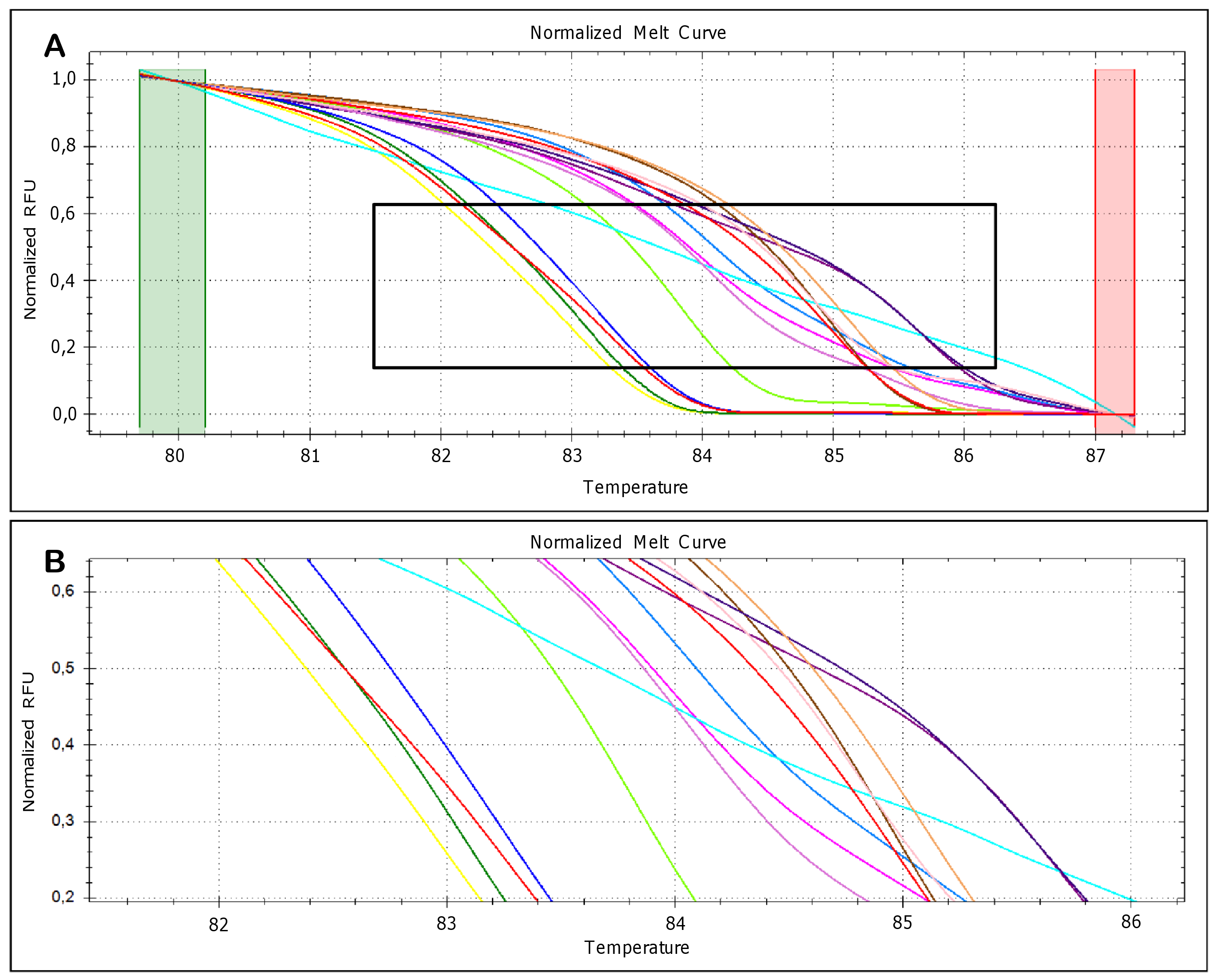

Supplement: Figure S5 — HRMA multiplex assay on bacteria related to P. savastanoi . (A) Normalized melting curves obtained by using as template pure DNAs (10 pg/reaction) extracted from bacteria closely related to P. savastanoi (Table S1), and the primer pairs for SNP markers C2/L1. Pure DNAs from Psv5, Psn23 and Psf134 were used as positive controls. Traces of different color indicate distinct HRMA profiles, produced by these bacteria and according to the color code reported in Table S1. No HRMA detectable signal was obtained for P. alcaliphila str. 28. and P. fluorescens str. 11. (B) Magnification of black-squared area in (A). (TIFF) [file pone.0030199.s005.tif]

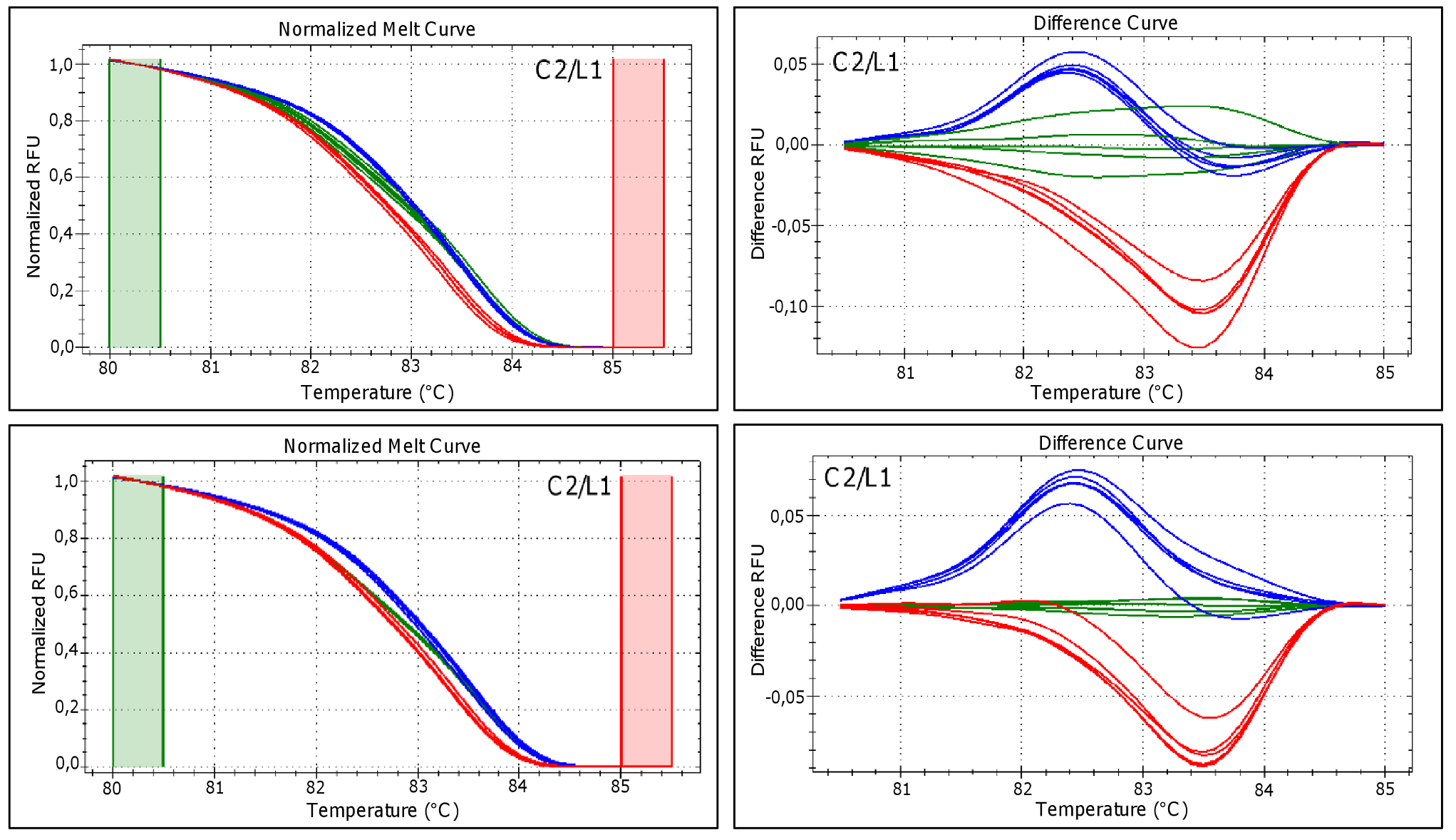

Supplement: Figure S6 — Specificity of multiplex HRM assay for P. savastanoi pathovars detection tested against Oleander epiphytes. Normalized and difference HRM plots obtained by multiplex HRM assay performed with the combination of primer pairs for SNP markers C2/L1. Washings from Oleander leaves artificially surface-inoculated with Psv5, Psn23 and Psf134 were used for thermal lysis extraction of DNA, to be used as template. Bacterial suspension drops of 5 µl (upper plots) and 50 µl (lower plots) were spotted on each leaf side. Pure genomic DNAs from Psv5, Psn23 and Psf134 were also tested as controls. On each plot red, green and blue traces were produced by Psv5, Psn23 and Psf134, respectively. RFU: Relative fluorescence units. Green and red columns represent pre- and post-melting normalization regions. (TIF) [file pone.0030199.s006.tif]
