# Supplementary material for: High-Resolution Melting Analysis as a Powerful Tool to Discriminate and Genotype Pseudomonas savastanoi Pathovars and Strains
Source: PLoS One. 2012 Jan 25;7(1):e30199. doi: 10.1371/journal.pone.0030199 (PMC3266268; doi:10.1371/journal.pone.0030199)
Supplement: Table S1 — Bacteria related to P. savastanoi or ubiquitous used in this study. (DOC) [file pone.0030199.s007.doc]

**Table S1 -** Bacteria related to *P. savastanoi* or ubiquitous used in this study.

| **Bacterial species and strains^a^** | **Color code^b^** |
| --- | --- |
| *P. syringae* NCPPB1053 |  |
| *P. syringae* pv. *apii* NCPPB1626 |  |
| *P. syringae* pv*. garcae* NCPPB2708 |  |
| *P. syringae* pv. *glycinea* NCPPB2753 |  |
| *P. syringae* pv. *maculicola* str. LPVM1 |  |
| *P. syringae* pv. *phaseolicola* NCPPB 2571 |  |
| *P. syringae* pv*. syringae* NCPPB2268 |  |
| *P. syringae* pv. *tagetis* NCPPB2488 |  |
| *P. syringae* pv. *tagetis* NCPPB2489 |  |
| *P. syringae* pv. *tomato* NCPPB2563 |  |
| *P. alcaliphila* str. 28 |  |
| *P. corrugata* str. 34 |  |
| *P. fluorescens* str. 11 |  |
| *Pantoea stewartii* str. 88 |  |
| *Pantoea stewartii* str. 89 |  |
| *Psv*5^c^ |  |
| *Psn*23^c^ |  |
| *Psf*134^c^ |  |

**^a^** NCPPB, National Collection of Plant Pathogenic Bacteria, York, UK (http://www.nctc.org.uk); *Pantoea* strains from culture collection of Laboratorio di Patologia Vegetale Molecolare, Dipartimento di Biotecnologie Agrarie, Università degli Studi di Firenze; *P. corrugata*, *alcaliphila* and *fluorescens* isolated and kindly provided by Dott. C. Viti, Dipartimento di Biotecnologie Agrarie, Università degli Studi di Firenze.

**^b^** Color code used to identify HRMA traces shown in Figure S5.

**^c^** *Psv5*, *Psn23* and *Psf134* were used as positive controls in HRMA experiment reported in Figure S5.
