# Supplementary material for: High-Resolution Melting Analysis as a Powerful Tool to Discriminate and Genotype Pseudomonas savastanoi Pathovars and Strains
Source: PLoS One. 2012 Jan 25;7(1):e30199. doi: 10.1371/journal.pone.0030199 (PMC3266268; doi:10.1371/journal.pone.0030199)
Supplement: Table S2 — Identification of epiphytic bacterial genera present on Oleander leaf washings using 16S rDNA gene sequencing, according to BLAST analysis. (DOC) [file pone.0030199.s008.doc]

**Table S2 -** Identification of epiphytic bacterial genera present on Oleander leaf washings using 16S rDNA gene sequencing, according to BLAST analysis.

| **Bacterial genera** | **clones (n°)** | **clones (%)** |
| --- | --- | --- |
| *Pseudomonas* spp*.* | 19 | 23 |
| *Xantomonas* spp*.* | 12 | 15 |
| *Bacillus* spp*.* | 11 | 14 |
| *Erwinia* spp*.* | 9 | 11 |
| *Acinetobacter* spp*.* | 7 | 9 |
| *Lactobacillus* spp. | 2 | 2 |
| Uncolturable bacteria | 21 | 26 |
